# *Borrelia miyamotoi* Infections in Humans and Ticks, Northeastern China

**DOI:** 10.3201/eid2402.160378

**Published:** 2018-02

**Authors:** Bao-Gui Jiang, Na Jia, Jia-Fu Jiang, Yuan-Chun Zheng, Yan-Li Chu, Rui-Ruo Jiang, Ya-Wei Wang, Hong-Bo Liu, Ran Wei, Wen-Hui Zhang, Yan Li, Xiao-Wei Xu, Jin-Ling Ye, Nan-Nan Yao, Xiao-Jing Liu, Qiu-Bo Huo, Yi Sun, Ju-Liang Song, Wei Liu, Wu-Chun Cao

**Affiliations:** Beijing Institute of Microbiology and Epidemiology, Beijing, China (B.-G. Jiang, N. Jia, J.-F. Jiang, R.-R. Jiang, Y.-W. Wang, H.-B. Liu, R. Wei, W.-H. Zhang, Y. Sun, W. Liu, W.-C. Cao);; Mudanjiang Forestry Central Hospital, Mudanjiang City, China (Y.-C. Zheng, Y.-L. Chu, Y. Li, X.-W. Xu, J.-L. Ye, N.-N. Yao, X.-J. Liu, Q.-B. Huo, J.-L. Song)

**Keywords:** *Borrelia miyamotoi* disease, bacteria, infections, human, tick, tickborne, vector-borne infections, China

## Abstract

We conducted an investigation of *Borrelia miyamotoi* infections in humans and ticks in northeastern China. Of 984 patients reporting recent tick bites, 14 (1.4%) were found to be infected with *B. miyamotoi* by PCR and genomic sequencing. The 14 patients had nonspecific febrile manifestations, including fever, headache, anorexia, asthenia, and arthralgia. Rash, eschar, and regional lymphadenopathy were each observed in 1 patient. Four (28.6%) patients were hospitalized because of severe disease. *B. miyamotoi* was detected in 3.0% (19/627) of *Ixodes persulcatus*, 1 (2.8%) of 36 *Haemaphysalis concinna*, and none of 29 *Dermacentor silvarum* ticks. Phylogenetic analyses based on sequences of a nearly entire 16s rRNA gene, a partial flagellin gene, and the glycerophosphodiester phosphodiesterase gene revealed that *B. miyamotoi* identified in patients and ticks were clustered in the group of the Siberian type. These findings indicate that *B. miyamotoi* is endemic in northeastern China and its public health significance deserves further investigation.

*Borrelia miyamotoi* is an emerging pathogen first identified in *Ixodes persulcatus* ticks and rodents from Japan in 1994 (*1**,**2*). Since then, this bacterium has been discovered in various other *Ixodes* tick species, including *I. ricinus*, *I. scapularis*, *I. pacificus*, *I. ovatus*, and *I. pavlovskyi*, all of which are known for biting humans (*3*). Human infection with *B. miyamotoi* was first reported in Russia in 2011 (*4*) and subsequently in the United States (*5**,**6*), Europe (*7**,**8*), and Japan (*9*). *B. miyamotoi* disease usually manifests as a febrile illness characterized by fatigue, headache, chills, myalgia, arthralgia, and nausea (*3**,**6*).

Human-biting ticks are highly prevalent in northeastern China, where various emerging tickborne infections have been found in ticks and humans (*10*). However, some febrile patients with a recent tick bite could not have infection with the known tickborne pathogen diagnosed. We conducted an investigation of *B. miyamotoi* infections in patients and questing ticks to understand the potential threat of this bacterium to humans in this region.

## Materials and Methods

### Patients and Data Collection

Patients reporting a recent tick bite and who saw a doctor at Mudanjiang Forestry Central Hospital during May 2013–June 2015 were enrolled in the study. The hospital is one of the largest in Mudanjiang City, located in Heilongjiang Province of northeastern China (*11*). We administered a standardized questionnaire to each participant to collect demographic information, medical history, and environmental exposures. We retrieved data on clinical manifestations, underlying conditions, laboratory tests, treatment, and outcomes from medical records. Blood samples were collected on the day when the patient saw a doctor. All participants provided written or oral informed consent.

### Tick Collection

**Figure 1:**
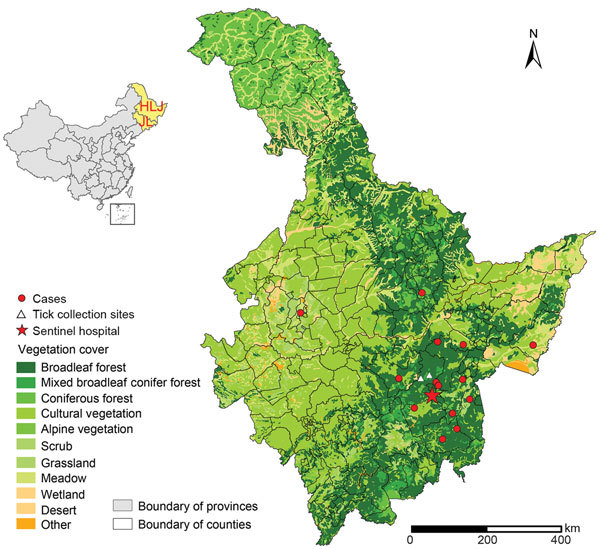
Geographic distribution of patients with *Borrelia miyamotoi* infection, northeastern China, May 2013–June 2015. Red dots (cases) indicate locations of case-patients’ residences. Inset map shows location of study area in China. HLJ, Heilongjiang Province; JL, Jilin Province.

During the study period, we collected questing ticks in the same region where the infected patients resided by dragging a flannel flag over vegetation. The 2 collection sites are forested highlands with elevations of 410 m and 550 m above sea level and harbor the same types of habitats as those areas where patients presumably were exposed to ticks (Figure 1). Approximately 20–35 ticks were collected per hour per person. All ticks were identified to the species level and developmental stage by an entomologist and stored at −20°C until DNA extraction.

### PCR Detection and Sequencing

We used 200 μL blood from each patient to extract DNA with the QIAmp DNA Blood Mini Kit (QIAGEN, Germantown, MD, USA) according to the manufacturer’s instructions. We ground each tick individually in a 200-μL phosphate-buffered saline buffer and extracted total DNA with the DNeasy Tissue Kit (QIAGEN). We used real-time PCR (rPCR) targeting of the 353-bp partial *B. miyamotoi* 16S rRNA gene (*rrs*) for screening blood and tick samples (*4*). The samples that were positive by rPCR were then subjected to amplification of a nearly full-length *rrs*, a partial flagellin gene (*fla*), and a partial glycerophosphodiester phosphodiesterase gene (*glp*Q) with specific primers (*12*; Technical Appendix Table 1). In addition, we tested all samples by PCR for spotted fever group rickettsiae (*13*), pathogens in the *Anaplasmataceae* family (*14*), *Borrelia burgdorferi* sensu lato (*15*), and *Babesia* (*11*). To avoid risk for contamination, we performed template isolation, PCR setup, and agarose gel electrophoresis in separate rooms, and a negative control (distilled water) was concurrently included in each amplification. All the PCR products were purified with the QIAmp Gel Extraction Kit (QIAGEN) and then directly sequenced on an automated DNA sequencer (3730 DNA Sequencer; Applied Biosystems, Carlsbad, CA, USA). We compared the sequences obtained with previously published sequences deposited in GenBank by using BLAST (http://blast.ncbi.nim.nih.gov/Blast.cgi).

### Phylogenetic Analyses

We conducted phylogenetic analyses based on nucleotide sequences of *rrs* (1,400 bp), *fla* (506 bp), and *glp*Q (461 bp) by using the maximum-likelihood method in MEGA software version 6.0 (http://www.megasoftware.net). We applied bootstrap analysis of 1,000 replicates to assess the reliability of the reconstructed phylogenies.

## Results

### Identification of *B. miyamotoi* Infection in Patients

**Figure 2:**
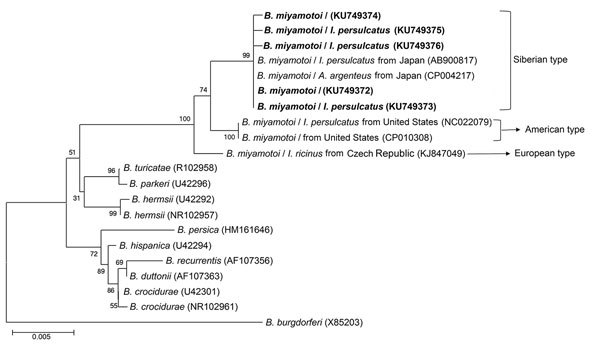
Phylogenetic tree based on nucleotide sequences of the 16s rRNA (1,400-bp) genes of *Borrelia miyamotoi* isolates from humans and ticks in northeastern China, May 2013–June 2015, and comparison sequences. Boldface indicates the *B. miyamotoi* identified in this study; GenBank accession numbers are provided for all isolates. Neighbor-joining trees were constructed by using the maximum-likelihood method in MEGA software version 6.0 (http://www.megasoftware.net). Scale bar indicates estimated evolutionary distance.

**Figure 3:**
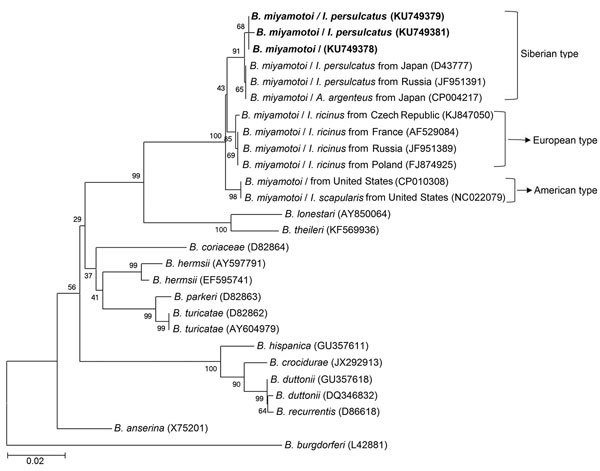
Phylogenetic analyses based on nucleotide sequences of the flagellin (506-bp) genes of *Borrelia miyamotoi* isolates from humans and ticks in northeastern China, May 2013–June 2015, and comparison sequences. Boldface indicates the *B. miyamotoi* identified in this study; GenBank accession numbers are provided for all isolates. Neighbor-joining trees were constructed by using the maximum-likelihood method in MEGA software version 6.0 (http://www.megasoftware.net). Scale bar indicates estimated evolutionary distance.

**Figure 4:**
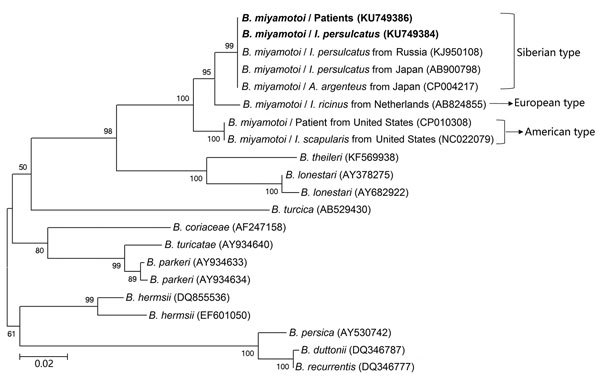
Phylogenetic analyses based on nucleotide sequences of the glycerophosphodiester phosphodiesterase (461-bp) genes of *Borrelia miyamotoi* isolates from humans and ticks in northeastern China, May 2013–June 2015, and comparison sequences. Boldface indicates the *B. miyamotoi* identified in this study; GenBank accession numbers are provided for all isolates. Neighbor-joining trees were constructed by using the maximum-likelihood method in MEGA software version 6.0 (http://www.megasoftware.net). Scale bar indicates estimated evolutionary distance.

During the study period, we screened a total of 984 participants who had a recent tick bite and sought medical care by using a rPCR assay specific for *B. miyamotoi*. Fourteen patients were positive. Nucleotide sequences of the 353-bp amplicons for all patients were identical to one another and to the corresponding fragment of *B. miyamotoi*. The nucleotide sequences of nearly the entire *rrs* were obtained from 5 patients (patients 2, 3, 7, 8, 13) (Table); 4 of these were identical to one another (GenBank accession no. KU749372) and to an FR64b strain from a rat in Japan. The other sequence had 1-bp difference (GenBank accession no. KU749374) (Figure 2). We amplified and sequenced partial *fla* (GenBank accession no. KU749378) and *glp*Q (GenBank accession no. KU749386) from 7 patients (patients 1, 2, 3, 7, 8, 9, 13) (Table); these sequences were 100% homologous to one another. The *fla* amplicons from all patients showed 1 bp difference with the corresponding sequence of the FR64b strain from Japan (Figure 3), whereas the *glp*Q amplicons had an identical sequence to that of FR64b (Figure 4).

**Table:** Epidemiologic, clinical, and laboratory testing features of 14 patients with *Borrelia miyamotoi* infections, northeastern China, May 2013–June 2015*

Characteristic	Patient no.													
1	2	3	4	5	6	7	8	9	10	11	12	13	14
Epidemiologic features														
Sex	F	M	F	M	M	M	M	M	M	F	M	M	M	M
Age, y	46	30	31	47	41	43	49	52	10	65	36	48	52	43
History of tick bite	Yes	Yes	Yes	Yes	Yes	Yes	Yes	Yes	Yes	Yes	Yes	Yes	Yes	Yes
Place of exposure to ticks	Forest	Forest	Forest	Forest	Forest	Forest	Forest	Forest	Home	Forest	Forest	Forest	Forest	Forest
Tick bite location	Arm	Trunk	Neck	Ear	Leg	Trunk	Trunk	Trunk	Scalp	Arm	Trunk	Trunk	Trunk	Trunk
Days from tick exposure to illness onset	18	28	16	5	3	7	7	25	4	10	26	6	4	40
Clinical manifestations														
Fever, °C	38.4	39.7	39	39	–	–	39.6	38.5	–	–	–	–	–	–
Asthenia	+	+	+	+	–	–	–	–	+	–	–	–	–	–
Headache	+	+	+	+	–	–	–	+	–	–	–	+	–	–
Anorexia	+	–	+	+	–	–	–	+	–	–	–	+	–	–
Myalgia	–	–	–	–	–	–	+	–	–	–	–	–	–	–
Arthralgia	–	+	+	–	–	–	+	–	–	–	–	–	–	–
Lymphadenopathy	–	–	–	–	–	–	–	–	+	–	–	–	–	–
Rash	–	–	–	–	–	–	–	–	–	+	–	–	–	–
Eschar	–	–	–	–	–	–	–	–	+	–	–	–	–	–
Laboratory findings†														
Leukocytes, 10^9^/L	7.9	9.6	8.9	11.7	5.1	NA	4.3	NA	9.9	7.6	NA	6.3	5.4	11.8
Lymphocytes, 10^9^ cells/L	0.9	0.6	0.9	1.8	1.7	NA	0.3	NA	3	2.2	NA	2	2.5	1.2
Neutrophils, 10^9^ cells/L	7.4	5.5	8.4	8.3	2.7	NA	3.9	NA	6	16.9	NA	4.2	2.7	9.7
Platelets, 10^9^/L	164	130	173	210	218	NA	79	NA	298	200	NA	172	137	269
AST, U/L	94.4	30.5	13.9	31	16.7	NA	60	NA	11.4	NA	NA	22	10.7	NA
ALT, U/L	124.6	17.8	12.1	26.1	24.3	NA	71.6	NA	8	NA	NA	39.5	16.2	NA
Hospital stay (d)	Yes (17)	Yes (3)	Yes (15)	Yes (20)	No	No	No	No	No	No	No	No	No	No
Co-infection	No	No	No	CRT	CRT	AC	No	No	No	No	No	No	No	No

*AC, *Anaplasma capra*; ALT, alanine aminotransferase; AST: aspartate aminotransferase; CRT, *Candidatus *Rickettsia tarasevichiae; NA, not applicable; +, positive; –, negative.  †Normal ranges: leukocyte count, 4–10 × 10^9^ cells/L; lymphocyte count, 0.8–4 × 10^9^ cells/L; neutrophil count, 2–7 × 10^9^ cells/L; platelet count, 100–300 × 10^9^/L; AST, 8–40 U/L; ALT, 5–40 U/L.

### Epidemiologic and Clinical Characteristics of the Patients

The 14 patients were distributed in 11 counties within the hospital catchment area (Figure 1). Ages ranged from 10 to 65 years (median 44.5 years), and 11 patients were male. All the patients had recent tick bites at various locations: 8 on the trunk, 3 on extremities, and 1 each on the scalp, ear, and neck (Table). Thirteen patients received a tick bite while they collected herbs in the forest, and 1 was bitten at home while taking care of goats that usually roamed in the forest during daytime. The median interval between known tick bite and illness onset ranged from 3 to 40 days (median 8.5 days). All 14 patients were immunocompetent and previously healthy.

The patients with *B. miyamotoi* infection had influenza-like manifestations, such as fever, headache, anorexia, asthenia, and arthralgia (Table). Rash, eschar, and regional lymphadenopathy were each observed in 1 patient. Elevated hepatic aminotransferase levels, increased leukocyte count, and thrombocytopenia occurred occasionally.

Four patients (patients 1–4) (Table) were hospitalized because of severe disease with an irregular fever up to 38.4°C–39.7°C. The median length of hospital stay was 14 days (range 3–20 days). Two hospitalized patients received doxycycline (100 mg 2×/d); the other 2 inpatients and 10 outpatients were not treated with doxycycline because their infections were diagnosed retrospectively. Defervescence occurred 3–5 days after treatment, and all the clinical manifestations disappeared subsequently. No in-hospital deaths or clinically significant sequelae were noted in follow-up observations. Two patients were co-infected with *Candidatu*s Rickettsia tarasevichiae (patients 4 and 5) and 1 with *Anaplasma capra* (patient 6). In comparison with the other 11 patients, the 3 patients co-infected with 2 tickborne pathogens had no more complicated symptoms or prolonged course of disease.

### Identification of *B. miyamotoi* in Ticks

We captured a total of 692 adult ticks: 627 *I. persulcatus*, 36 *H. concinna*, and 29 *D. silvarum*. We detected *B. miyamotoi* in 19 (3.0%) *I. persulcatus*, 1 (2.8%) *H. concinna*, and no *D. silvarum* ticks (Technical Appendix Tables 2, 3). We then amplified the nucleotide sequences of nearly the complete *rrs* from 7 *I. persulcatus* ticks. Five of these were identical to one another (GenBank accession no. KU749373) and to those from 4 of 5 human patients tested (GenBank accession no. KU749372); however, the 7 ticks had 1-bp difference with the other 2 *I. persulcatus* ticks (GenBank accession nos. KU749375 and KU749376) and the other patient (GenBank accession no. KU749374) (Figure 2). The *fla* sequences from 6 *I. persulcatus* ticks were 100% homologous (GenBank accession no. KU749379) to those of patients; 1 sequence (GenBank accession no. KU749381) was different by 2 bp (Figure 3). The sequences of *glp*Q from the 8 *I. persulcatus* ticks (GenBank accession no. KU749384) were exactly the same as each other and those from patients (Figure 4).

### Genetic Characteristics of *B. miyamotoi*

The phylogenetic trees based on nearly the complete *rrs* (1,400 bp), the partial *fla* (506 bp). and the partial *glp*Q (461 bp), sequences demonstrated that the *B. miyamotoi* identified from patients or ticks in this study were clustered in the same clade as those from Japan and Russia belonging to the Siberian type, which was distinct from both European and American types (Figures 2–4).

## Discussion

The *B. miyamotoi* bacterium is a newly described emerging pathogen, which was known to be transmitted to human beings by *Ixodes* ticks in North America and Eurasia (*6**,**16*). Since the first identification of human infections in Russia in 2011, patients infected with *B. miyamotoi* have been sequentially diagnosed in the United States, Europe, and Japan (*6*). In this study, we report *B. miyamotoi* infections in a series of patients and in *I. persulcatus* and *H. concinna* ticks in northeastern China. These findings imply that persons who are exposed to ticks might run a risk of contracting *B. miyamotoi* disease. The dispersed geographic distribution of infected patients (Figure 1) indicates that *B. miyamotoi* might be widely prevalent in this region.

Although no clinically validated test is available for *B. miyamotoi*, the disease can be diagnosed by PCR performed on blood during acute-phase infection through amplifying and sequencing the *rrs*, *fla*, and *glp*Q genes. These PCRs are effective methods because of their high sensitivity and specificity for detecting *B. miyamotoi* DNA in either blood or tick samples, and they provide a valuable tool not only for the diagnosis of human infections but also for identification of *B. miyamotoi* in ticks (*3**,**6*). In this study, we used rPCR for detecting the 16S rRNA gene of *B. miyamotoi* in human and ticks. The sensitivity of the assay was reported to be up to 5 × 10^3^ copies/mL as determined by using recombinant DNA of the *B. miyamotoi* 16S rRNA gene fragment with a known number of copies (*4*). We then sequenced all the 353-bp amplicons of rPCR to confirm the specificity of the test. The samples positive for rPCR were subjected to amplification of the nearly complete *rrs* and partial *fla* and *glp*Q genes. The phylogenetic analyses based on the 3 genes revealed that *B. miyamotoi* from patients and ticks were genetically homogeneous to one another and clustered in the same group with those from Japan and Russia, belonging to the Siberian type (*3**,**4**,**6*).

Human cases with *B. miyamotoi* infection have been diagnosed in Russia, the United States, European countries, and Japan (*4**–**9*). These reports imply its public health significance, given that human *B. miyamotoi* infection appears to be comparable in frequency to human granulocytic anaplasmosis and babesiosis in some areas (*6**,**17*), and can occasionally lead to severe illness with neuroborrelosis and meningoencephalitis, especially in immunocompromised persons (*5**,**8**,**18*). The most commonly reported clinical manifestations of *B. miyamotoi* infection are fever, fatigue, headache, chills, myalgia, arthralgia, and nausea (*6*). The 14 patients in our study were immunocompetent and had similar manifestations observed in the previously reported cases in other countries (Table). Four patients (28.6%) were hospitalized for severe illness. *B. miyamotoi* infection should be included in the differential diagnosis of patients with a history of tick bite in areas where this pathogen has been identified in ticks or humans.

Up to now, *B. miyamotoi* has been found only in *Ixodes* ticks from wide geographic range, including Japan, Russia, the United States, Canada, and many countries in Europe, including the Czech Republic, Denmark, England, Estonia, France, Germany, the Netherlands, Poland, Sweden, and Switzerland (*6**,**17**,**19**,**20*). In this study, we detected *B. miyamotoi* not only in *I. persulcatus* ticks but also in *H. concinna* ticks captured in the patients’ residences. This finding indicates that both *Ixodes* and *Haemaphysalis* tick species might serve as competent vectors of *B. miyamotoi*. Unfortunately, we did not collect or record the species of the ticks attached to the patients who were seen at the hospital during the study period (May 2013–June 2015). According to our 2016 surveillance data, 93 of the 511 patients in the same sentinel hospital saw a doctor while the tick was still attached. These ticks were 80 (86.0%) *I. persulcatus*, 5 (5.4%) *H. concinna*, and 8 (8.6%) *D. silvarum,* roughly reflecting the abundance of the 3 tick species in the environment.

In conclusion, physicians should be aware of the presence of *B. miyamotoi* in northeastern China and make the differential diagnosis for patients with an exposure to ticks to attain an etiologic testing and effective treatment. The public health significance as well as potentially wider distribution of the emerging disease deserves further investigation.

Technical Appendix Sequence amplification used in detection of *Borrelia miyamotoi* infection, nucleotide sequences of primers used in the study, and prevalence of *B. miyamotoi* infection in ticks, northeastern China, May 2013–June 2015.
