# Supplementary material for: Borrelia miyamotoi Infections in Humans and Ticks, Northeastern China
Source: Emerg Infect Dis. 2018 Feb;24(2):236–41. doi: 10.3201/eid2402.160378 (PMC5782893; doi:10.3201/eid2402.160378)
Supplement: Technical Appendix — Sequence amplification used in detection of Borrelia miyamotoi infection, nucleotide sequences of primers used in the study, and prevalence of B. miyamotoi infection in ticks, northeastern China, May 2013–June 2015. [file 16-0378-Techapp-s1.pdf]

Article DOI: https://doi.org/10.3201/eid2402.160378

Borrelia miyamotoi Infections in Humans and Ticks, Northeastern China

Technical Appendix

Sequence Amplification of *Borrelia miyamotoi* Infection

Primer Brm1/Brm2 was used for real-time PCR screening of *B. miyamotoi* (353-bp) infection, the positive representative melting temperature peaks in 86 ±0.5°C. All the positive outcomes (353-bp) were sequenced. For every positive specimens, primer Bf1 (or rrs1F)/rrs4R was used as nested-PCR outer primers, Bf1 (or rrs1F)/BmR and BmF/rrs4R were used as inner primers for amplification front and back fragments of *rrs* gene respectively, splicing with middle fragments (Brm1/Brm2, 353-bp), eventually a 1499-bp or 1523-bp fragments for *rrs* gene was acquired based on overlapping sequence. Primer FLA120F/FLA920R was used as nested-PCR outer primers, FLA120F/flab625R as inner primers for amplification *fla* gene partial sequences (506-bp). Primer glpQ67F/glpQ789R was used as nested-PCR outer primers, glpQ67F (or glpQ86F) /glpQ546R as inner primers for amplification *glpQ* gene partial sequences (480-bp or 461-bp).

Technical Appendix Table 1. Nucleotide sequences of the primers used in the study

Target gene	Primer	Sequence (5′-3′)	Position*	Objective	Reference
rrs	Brm1	CGCTGTAAACGATGCACACTTGGTGTTAATC	807F	Screening of *B. miyamotoi* (353-bp)	(*1*)
	Brm2	CGGCAGTCTCGTCTGAGTCCCCATCT	1159R		(*1*)
	Bf1	GCTGGCAGTGCGTCTTAAGC	38F	Amplification and splicing *rrs* complete sequence	(*1*)
	rrs1F	GTTTGATCCTGGCTTAGAAC	14F		(*2*)
	rrs4R	GAGGTGATCCAGCCACACTTT	1536R		(*2*)
	BmF	AGATGGGGACTCAGACGAGACTGCCG	1134F		This study
	BmR	GATTAACACCAAGTGTGCATCGTTTACAGCG	837R		This study
fla	FLA120F	AGAATTAATMGHGCWTCTGATGATG	103F	Amplification *fla* partial sequence (506-bp)	(*1*)
	FLA920R	TGCYACAAYHTCATCTGTCATT	912R		(*1*)
	Flab625R	CTGGAGCTGCTTGAGCACCTTCT	622R		This study
glpQ	glpQ67F	ATGGGTTCAAACAAAAAGTCACC	67F	Amplification *glpQ* partial sequence (461-bp)	This study
	glpQ86F	CACCATTRATYATAGCTCACAG	86F		This study
	glpQ546R	GATGTCTTTACCTTGTTGTTTATGCCA	546R		This study
	glpQ789R	CCAGGGTCCAATTCCATCAGAATATT	789R		This study

*F, forward direction; R, reverse direction. Primers position are referring to the corresponding sequence of *rrs, fla*, and *glpQ* of *B. miyamotoi*.

Technical Appendix Table 2. *Borrelia miyamotoi* infection prevalence in ticks

	No. *B. miyamotoi*–positive ticks (%)		
Tick species	Male	Female	Total
I. persulcatus	13/343 (3.8)	6/284 (2.1)	19/627 (3.0)
H. concinna	1/12 (8.3)	0/24 (0)	1/36 (2.8)
D. silvarum	0/13 (0)	0/16 (0)	0/29 (0)
Total	14/368 (3.8)	6/324 (1.9)	20/692 (2.9)

A total of 692 adult ticks including 627 (90.6%) *I. persulcatus*, 36 (5.2%) *H. concinna*, and 29 (4.2%) *D. silvarum*.

Technical Appendix Table 3. *Borrelia miyamotoi* gene amplification in ticks

No.	Year	Tick ID	Collection site	Tick species	Tick sex	*rrs* partial	*rrs* complete	*fla* partial	*glpQ* partial
1	2013	101	E44°55′, N129°30′	*I. persulcatus*	Female	353 bp	NA.	NA	NA
2	2013	105	E44°55′, N129°30′	*I. persulcatus*	Female	353 bp	NA.	NA	NA
3	2013	119	E44°55′, N129°30′	*I. persulcatus*	Male	353 bp	NA.	NA	NA
4	2013	176	E44°55′, N129°30′	*H. concinna*	Male	353 bp	NA.	NA	NA
5	2013	237	E44°55′, N129°30′	*I. persulcatus*	Female	353 bp	NA.	NA	NA
6	2013	350	E44°58′, N129°13′	*I. persulcatus*	Female	353 bp	1499 bp,	506 bp	461 bp
7	2013	392	E44°58′, N129°13′	*I. persulcatus*	Male	353 bp	1499 bp	506 bp	461 bp
8	2013	421	E44°58′, N129°13′	*I. persulcatus*	Male	353 bp	NA.	NA	NA
9	2013	428	E44°58′, N129°13′	*I. persulcatus*	Male	353 bp	1499 bp	506 bp	461 bp
10	2013	544	E44°58′, N129°13′	*I. persulcatus*	Male	353 bp	1499 bp	506 bp	461 bp
11	2013	606	E44°55′, N129°30′	*I. persulcatus*	Male	353 bp	1499 bp	NA	461 bp
12	2013	609	E44°55′, N129°30′	*I. persulcatus*	Male	353 bp	NA.	NA	NA
13	2013	622	E44°55′, N129°30′	*I. persulcatus*	Male	353 bp	NA.	NA	NA
14	2014	1	E44°58′, N129°13′	*I. persulcatus*	Male	353 bp	NA.	NA	NA
15	2014	52	E44°58′, N129°13′	*I. persulcatus*	Female	353 bp	1499 bp	506 bp	461 bp
16	2014	114	E44°55′, N129°30′	*I. persulcatus*	Male	353 bp	1499 bp	506 bp	461 bp
17	2014	121	E44°55′, N129°30′	*I. persulcatus*	Male	353 bp	NA.	NA	NA
18	2014	124	E44°55′, N129°30′	*I. persulcatus*	Male	353 bp	NA.	NA	NA
19	2014	126	E44°55′, N129°30′	*I. persulcatus*	Male	353 bp	NA.	NA	NA
20	2014	150	E44°55′, N129°30′	*I. persulcatus*	Female	353 bp	NA.	506 bp	

References

1. Hovius JW, de Wever B, Sohne M, Brouwer MC, Coumou J, Wagemakers A, et al. A case of meningoencephalitis by the relapsing fever spirochaete *Borrelia miyamotoi* in Europe. Lancet. 2013;382:658. PubMed http://dx.doi.org/10.1016/S0140-6736(13)61644-X

2. Potkonjak A, Kleinerman G, Gutiérrez R, Savić S, Vračar V, Nachum-Biala Y, et al. Occurrence of *Borrelia burgdorferi* sensu lato in *Ixodes ricinus* ticks with first identification of *Borrelia miyamotoi* in Vojvodina, Serbia. Vector Borne Zoonotic Dis. 2016;16:631–5. PubMed http://dx.doi.org/10.1089/vbz.2016.2008
